# Expanded-multidimensional turnover intentions: scale development and validation

**DOI:** 10.1186/s40359-023-01303-2

**Published:** 2023-09-11

**Authors:** Obinna Osita Ike, Lawrence Ejike Ugwu, Ibeawuchi K. Enwereuzor, Ifeanyichukwu Chukwudi Eze, Obiageli Omeje, Ejike Okonkwo

**Affiliations:** 1 Psychology Department, University of Nigeria, Nsukka, Nigeria; 2 Psychology Department, Psychology Department, Renaissance University Ugbawka Enugu, Enugu, Nigeria; 3 Psychology Department, Enugu State University of Science and Technology, Enugu, Nigeria; 4 Police Academy Wudil, Kano, Kano Nigeria

**Keywords:** Multidimensional turnover intentions, Scale development, Subjective social status, Organisational culture, Personal orientation, Expectations, Career growth

## Abstract

**Background:**

This study aims to provide researchers and practitioners with a more elaborate instrument to measure turnover intentions based on the planned behaviour theory model. The questionnaire assesses 5 distinct aspects of turnover intentions (i.e., subjective social status, organisational culture, personal orientation, expectations, and career growth).

**Methods:**

In this cross-sectional study (comprise of 2 studies in one) a wave survey design was applied to a large diversity of workers drawn from the staff of universities, banks, hospitals, factories, and telecommunication companies. Exploratory factor analysis (EFA) was applied the identify the sub-dimensions and Cronbach’s alpha to assess the reliability of the first study. In the second study, for the Confirmatory factor analysis to establishing structural model of the dimensions.

**Results:**

We demonstrate the reliability, factor structure, and validity evidence based on internal structure and relationship with other variables of the new measure among two samples (N_1_ = 622; N_2_ = 433). Twenty-five items with 5 factors were extracted to represent a broader perspective of turnover intention scale.

**Conclusions:**

In total, the study indicates that the assessment can be used to reliably assess several major indicators of turnover intentions. Therefore, improved employees’ evaluations and reduced loss of valuable staff as a result of avoidable measures in considering the interests of workers.

Turnover has attracted substantial scholarly attention in recent decades because of its practical significance [1], theoretical importance [2], and implications. Turnover intentions have been posited as the wilfulness to leave or quit one’s current organisation. Turnover has been classified as voluntary or involuntary; both can be considered planned behaviour [3]. It is voluntary when the employee plans when they feel dissatisfaction or the availability of an alternative. This costs the organisation a lot as they are challenged to get a suitable replacement. While involuntary, as planned by the organisation, is due to the organisation’s dissatisfaction with the employees’ services and must have prepared for a suitable replacement or an alternative arrangement [4]. This study focuses on voluntary turnover. The unexpected nature of this turnover affects organisations as they are usually caught off guard [5].

Many studies have highlighted possible reasons employees embark on voluntary turnovers, such as the availability of numerous job options, pay satisfaction, lack of motivation, inadequate working conditions, etc. (e.g [6, 7]).

Numerous researchers have tried to propose a theoretical explanation for organisational turnover based on [8], Mobley’s model of 1977, and [9], which all point to the satisfaction and dissatisfaction evaluation of employees’ current jobs. The model by [10] and job embeddedness by [11] contributed immensely to understanding turnover. Turnover has proven extremely difficult to measure due to the questions about its authenticity. Instead, the closest means to measure turnover is the intention of the turnover method. Numerous studies have demonstrated turnover intention as one of the most significant predictors of subsequent voluntary turnover behaviour (e.g [12, 13]).

## Why a new measure for turnover intentions?

The multitude of reasons why there is turnover intention has been over-simplified to a unidimensional scale, for example [14], three-item scale [15], two-item scale [16], five-item scale [11], three-item scale [17], six-item scale, and [18] eighteen-item scale. The absence of dimensionality in this research was recently redeemed by the seminal work of [5], who impressively developed a two-dimensional structure of turnover intentions (extrinsic and intrinsic perspectives). This effort cannot be said to have broadened perspectives of turnover intention rather, it has ignited more desire to search for more justification for turnover intention than just the extrinsic and intrinsic viewpoints.

In the effort not to oversimplify the decision to leave an organisation to lie on the comparison between the current organisation and another or the individual’s intrinsic desire but to identify key areas concerning the organisation and within the individual. Those key factors need to be weighed on its validity in the worker’s decision-making that suppresses them.

The methodological limitations of previous measures include poor psychometric properties (e.g., reliability and validity, too few items and sample size, limited context, etc.). Menezes and colleagues [5] acknowledged the possible administration biases and sample size challenges. Building on their efforts, this study further explained reasons for employee turnover intentions. To develop a more comprehensive, validated measurement of turnover intentions, the Expanded Multidimensional Turnover Intentions Scale (EMTIS) was developed to examine subjective social status (SSS), expectation, career growth, organisational culture, and Personal orientation factors that trigger the willingness to quit.

## Identifying dimensions of turnover intentions

### Subjective social status

Subjective social status (SSS) is one probable sign of turnover intention. It is defined as a person’s view of their place within a social structure [19]. Subjective social status is based on objective socioeconomic status indicators, such as occupation and income. Singh-Manoux and colleagues [20] posited that individuals could also employ factors like respect and reputation to determine their SSS [21]. explained that social status is an individual’s ability to control resources and represents positions in society and culture. Subjective Social status is negatively related to turnover intention [22, 23]. This relationship from studies connotes that those jobs with perceived high prestige, security, income, etc., decrease the tendencies of the worker to quit [22]. Social status might not be related to turnover intention in a job or some jobs or people within a location where options are limited, for example, in developing countries [24]. Other studies have opined that turnover can occur when employees wish to seek higher pay and social status [25–27]. Lower subjective social status was associated with different dimensions of poorer indications of good health (diabetes, respiratory challenges, angina issues etc.). Modification for indicators of objective social status (such as employment criteria and salaries) reduced the relationships such that only the relationship between lower subjective social status and perceived poor health remained strong [28]. The perceived lower subjective social status of the current position could trigger the intention to quit n.

### Organisational culture factors

Organisational culture is perceived as a company asset that can be used to increase business performance and influence work attitudes [29]. Organisational culture is a set of values, beliefs, and attitudes among members of the organisation [30]. The relationship between organisational culture and turnover intentions has been reported to have a mixed report of positive and negative relationships [31–34]. It is common knowledge in the research of turnover intention that it ranges between thoughts of leaving and the action of leaving and is considered the most important variable preceding actual turnover [12]. Turnover intention is affected by organisational culture factors such as job stress, organisational culture, and leadership system. Job stress has to do with the psychological and physiological consequences of people’s emotional response to stimuli from work exhaustion and anxiety from one’s job [35–37]. and [38] found that a high level of work-related stress and emotional drain not only reduces job satisfaction but also induces a turnover reaction. Kim and Han [39] identified four dimensions of organisational culture: relation, innovation, task, and hierarchy. Relation-oriented organisational culture is considered to focus on human relationships such as trust, participation teamwork, loyalty, and work morale. While the innovation-oriented organisational culture show-cased organisational change and inventiveness aimed at customer satisfaction, recognition of creativity, and entrepreneurship. Task-oriented organisational culture stresses productivity in achieving organisational performance and carrying out tasks and values external orientation and stability. Finally, hierarchy-oriented organisational culture highlights stability, ensuring respect for order or processes of doing things among members of the organisation. Organisations vary in the types of services they render, but one thing common among them is that they operate under a leader or leadership style. These styles can help the employees navigate the complexities with some ease. In contrast, others make it toxic and inject negative feelings that affect employees’ loyalty, productivity, motivation, health, and happiness [40–42]. Previous studies found that an excessively stressful environment may cause negative behaviours, leading to a focus on how system dynamics and organisational culture play a role in exhibiting toxic behaviours [43]. Doty and Fenlason [44] found that toxic behaviour in healthcare institutions was exhibited by narcissist leadership. Taştan [45] posited toxic conditions: toxic workplace, abusive management, and mobbing. The feeling of toxicity in one’s workplace and the inability to cope or adjust to them could make one plan to quit or search for a better alternative.

### Personal orientation factors

Personal orientation factors such as health problems, family-related issues (e.g., distance from family, marriage, etc.), age, child-rearing considerations, and well-being contribute to turnover intentions. However, very little empirical research is available on personal orientation-related factors [46, 47].

In an elaborate study by [48], she found that Personal orientation factors (e.g., work-family life, job satisfaction, general health, and organisational commitment significantly influence workers’ turnover intention. The personal connection people have with their occupations has gained significant research that indicated the critical emotional connection employees have with their profession (e.g [49–51]), It has been found that employees are also significantly committed to external factors (such as organisations). They also create significant links to personal passions that clearly connect with work values and beliefs (e.g [52, 53].,). Strong affection towards a job is often keeps and motivates employees in what many as a complex role [49]. Passion, social support, and self-centred leadership were found to relate indirectly to turnover intention through the full mediation of career commitment [54].

### Expectations

Drawing from psychological contract breach theory, expectations are based on the norm of reciprocity between employees and employers. The concept of the psychological contract, as promoted by [55], states that a psychological contract is an agreement, formal or informal, overt or implied, between two or more agents. This concept is described as relational, transactional, or hybrid. The direct and transparent record of duties, deliverables, compensation, and duration agreed upon by all parties is considered a transactional contract. While the indirect, informal, and vague agreements suggest that mutual emotional and physical investments exist called relational contracts. The hybrid contract shows elements of both relational and transactional contracts. A violation of the expectations of the implied or non-explicit agreements would be considered a breach. This violation is considered a good ground for turnover [56]. Workers and their employers have expectations of reciprocity, which is the belief that the organisation will provide rewards that match the workers’ effort. However, one of the challenges is covering workers’ expectations of reciprocity and recognising unmet expectations [57]. At the same time, expectations of reciprocity are prevalent in the psychological contract literature [58]. Arasli and colleagues [59] noted that the absence of a meritocratic system of promotion in the workplace breeds unjust and unfair practices, increasing turnover. Adopting favouritism when workers have been informed of a merit-based system constitutes a psychological contract violation [60]. Also, unclear roles have been found to significantly promote turnover intentions among workers, for example, nurses who are uncertain about expectations and requirements associated with their roles [61]. Yang and colleague [62] posited that in the event of an expectation violation, some employees would rather quit than adjust to the organisation as a means to cope. They suggested that this is acquiring new resources in another organisation rather than conserving existing resources (meaning adjusting in their current organisation). This factor expressly addresses the need for more research and method of measuring employees’ expectations of professional turnover.

An organisation’s career growth opportunities can be considered benefits in the Social Exchange Theory [63]. When employees perceive that their organisation offers ample opportunities for career advancement and growth, they may be more committed and loyal, as the perceived benefits (e.g., job satisfaction, skill development, promotion) outweigh the costs (e.g., time and effort) [24, 64, 65]. Organisations fostering a mutual-investment relationship with their employees create a more stable balance in the social exchange, leading to lower turnover rates and increased commitment.

### Career growth

Career growth is the extent to which an individual’s perception that his/her organisation provides opportunities, support, and an enabling environment where his or her career goals can be attained, which can lead to increased commitment and lower turnover rates [66, 67]. Previous studies have found that organisations enable their employees to achieve long-term career growth, encourage higher organisational commitment [66–68], and engage in extra-role behaviour (e.g [69, 70]). Tsui and colleagues [71] noted that organisations that create opportunities for career advancement have been shown to create a ‘mutual-investment’ relationship with their employees, which has, in turn, been found to be connected to outcomes of low turnover (e.g [68]).

#### Initial item generation and reduction

To establish reference point dimensions of Expanded Multidimensional turnover intentions, the researchers generated an initial item pool of 61 items from reviews of academic literature and existing constructs (for example, subjective social status [19]). Items that were too vague and unrelated to the Expanded Multidimensional turnover intention context were removed from the pool. As a result, 57 items were retained as a pilot set. These items were rephrased to reflect the characteristics of the Expanded Multidimensional turnover intention context.

six focus groups composed of five participants (in each) recruited from different work sectors (telecommunication, banking/finance, health, factory workers, academics, and administration) were conducted on separate days, in person and virtual, to fit into some of the participants’ schedules, to examine the readability of items, remove redundant and vague items in the initial set, and create new items. Participants were asked to recall their latest Expanded Multidimensional turnover intentions, indicate their level of agreement on the 57 items, and comment on the readability of the items. To ensure that the authors’ conceptualisations of Expanded Multidimensional turnover intentions were consistent with those of the workers, participants were also invited to give details on their reasons for Expanded Multidimensional turnover intentions.

The findings of the focus groups advocated that all the items were easily understood. Twelve items were rephrased better to reflect engagement patterns in Expanded Multidimensional turnover intentions. The participants’ answers could be accommodated within the five existing dimensions, and no new dimension emerged during the process.

An exploratory survey was conducted within five sectors (education, finance, health, industry, and telecommunication) to refine the items further. Survey respondents were recruited by distributing questionnaires in two Nigerian universities, four banks, two teaching hospitals, five factories, and four telecommunication companies. The items were reviewed by human resources management and psychology experts with doctoral qualifications to check the content validity. Seventeen experts were contacted, and ten agreed to participate in the study. Each expert was also asked to indicate their level of agreement with the 57 items and to provide comments and suggestions on the five Expanded Multidimensional turnover intention dimensions and the scale. Using [72] content validation method to evaluate the items based on relevance, clarity, and simplicity by responding to the 4-point Likert scale (1 = not relevant, 2 = somewhat relevant, 3 = quite relevant, 4 = highly relevant). Items with low content validity index of 0.83 were removed. This led to sixteen items being removed from the scale during this process.

## Stage 1: initial construct development

### Method

#### Participants

The participants for this study comprised six hundred and twenty-two (622) workers drawn from the staff of universities, banks, hospitals, factories, and telecommunication companies. The participants comprise both married and unmarried workers, male and female workers who have spent at least one year in the organisation, with at least SSCE/WAEC, and between the ages of eighteen and sixty.

The distribution of the sampled workers was as follows: - male 354 (56.9%), female 268 (43.1%), married 321 (51.6%), single 301(48.4%), supervisors 78 (12.5%), other workers 544 (87.5%). The participants ranged from 18 to 60 years with a mean age of 33.75 (M = 33.75, SD = 9.30). The educational level of the participants ranged from Senior School Certificate Holders (*n* = 85, 13.7%), Ordinary National Diploma (*n* = 206, 33.0%), Higher National Diploma (*n* = 161, 26.0%), Bachelor’s degree holders (*n* = 114, 18.3%), Master’s degree holders (*n* = 11, 1.8%) and doctoral degree holders (*n* = 45, 7.2%).

#### Instrument

Expanded multidimensional Turnover Intention Scale (EMTIS). The 41-item scale is measured on a five-point Likert scale, with anchors ranging from ‘strongly disagree’ to ‘strongly agree’. All the items were positively worded, with an overall maximum score of 205 and a minimum of 41.

#### Procedure

First, the researchers obtained approval and an introduction letter from the department. With this letter, the researchers obtained permission from the management of the participating organisations. The researchers recruited and trained four research assistants to assist in distributing and collecting copies of the questionnaire. All participants were informed that their responses to the questionnaire had no consequence or connection with the management and would remain confidential and voluntary. Eight hundred (800) copies of the questionnaire were distributed with the help of the research assistants. However, it took three working days for most of the copies of the questionnaire to be collected with the help of the research assistants in each of these organisations. After completion and collection, properly filled copies of the questionnaire were analysed. A total of six hundred and forty-eight (648) were returned, and twenty-six (26) copies were discarded due to improper completion. In contrast, six hundred and twenty-two (622) valid copies were used for the analysis, yielding a valid response rate of 77.8% out of eight hundred (800) copies of the questionnaire that were initially distributed.

#### Design/statistics

This pilot study adopted a cross-sectional survey design. This is because more samples were drawn from the population to elicit people’s responses to the variables of interest [73]. An exploratory factor analysis (EFA) was performed using the IBM Statistical Package for the Social Science (SPSS) version 25. While SmartPLS v 3.3 was also used to determine a clearer understanding of the individual parameters. Construct validity for example, Cronbach alpha (CA), composite reliability (CR), and average variance extracted (AVE).

### Result

A principal axis factor analysis (PAF) was carried out on the 41 items with direct oblimin rotation. This was done based on the assumption that the factors in the scale should correlate [74]. Items with cross-loadings above 0.40, factor loadings less than 0.50 and communalities less than 0.40 were removed from the pool [75]. Based on the factor analysis iteration process and item deletion, 16 items were excluded, and a 25-item inventory was retained. The item refinement study yielded a five-factor solution, accounting for 58.26% of the common variance. The factorial dimensions aligned with the authors’ theoretical conceptualisation of Expanded Multidimensional turnover intentions and were labelled as subjective status, organisational culture, personal orientation, expectation, and career growth, respectively. The Kaiser–Meyer–Olkin measure of sampling adequacy (MSA) index of 0.94 and a significant chi-square value for Bartlett’s test of sphericity less than 0.001 suggested the EFA is appropriate for the data. Cronbach’s alpha values for the Expanded Multidimensional turnover intentions dimensions ranged from 0.82 to 0.93, representing satisfactory reliabilities [75]. The remaining set of items used for confirmatory factor analysis (CFA) is presented in Table 1.

Table 1 demonstrates the statistical significance of each observation variable concerning its individual latent variable factor loading (λ).

All factors had values of 0.50 or higher, indicating that the observed variable reflected its construct’s latent variable. Factors with a CA and CR above 0.63 were considered satisfactory [76], and all factors exceeded 0.63. The AVE were all above the 0.50 endorsed threshold [77].

**Table 1:** Construct validity

Items	Factor Loading	CA	Rho_A	CR	AVE
**Subjective social status**		**0.903**	**0.904**	**0.932**	**0.774**
I do not like the image of me I see in the future if I remain here	0.845				
My present job leaves me no choice but to look for alternative job offer that will befit my status.	0.890				
I often feel like quitting this job because my present job position is not compatible with my job resume.	0.915				
I feel like quitting this job because of my marital status.	0.868				
**Organisational culture**		**0.816**	**0.841**	**0.890**	**0.729**
I often feel like staying at home than going to work because of the way my organisation is structured.	0.820				
I am seriously considering quitting this job because of the organisational practices and policies.	0.895				
My major dissatisfaction in life comes from my job environment.	0.844				
**Personal orientation**		**0.934**	**0.940**	**0.945**	**0.684**
Leaving my present job is my ultimate priority now because of family demand.	0.818				
My family is not happy with the nature of my job.	0.792				
I often consider leaving my job as a result of my health status.	0.837				
I cannot be fit enough to continue this job in the near future	0.876				
I often feel like quitting this job because the organisation does not keep to its promise.	0.861				
Most of people whose opinions I respect think I should leave my job.	0.796				
I intend to leave this organisation in the next one year.	0.848				
I often feel like quitting this organisation because I see no future in it.	0.785				
**Expectation**		**0.911**	**0.917**	**0.933**	**0.738**
Healthcare package is so poor to compare to the kind of work I do.	0.858				
If I get better offer, I will leave my present job because of job insecurity.	0.880				
I often feel that my present job is not worth the offer.	0.907				
Regardless of the pay, I would prefer working where I will be respected and recognized.	0.785				
What is holding me in this job is that I have not gotten an acceptable alternative offer/job that is lucrative.	0.859				
**Career Growth**		**0.927**	**0.932**	**0.945**	**0.776**
I often feel like quitting this organisation because my years of service do not reflect my present job designation.	0.833				
I want to learn few things concerning my job career in this organisation and leave.	0.841				
I know I deserve a better job, I will go for it when I find one	0.923				
I need a work environment that will improve me, I don’t get it here.	0.910				
I feel like quitting this organisation because it does not create opportunity for advancement and development.	0.893				

Note: CA= Cronbach Alpha, CR= composite reliability; AVE= Average Variance Extracted

## Stage 2: construct validation

Another set of data was collected to ascertain construct validity for the measurement. Convergent and discriminant validity were equally conducted in this second study.

The need for convergent validity is important to measure whether the earlier validated instrument for turnover intentions is related to the current instrument [78]. Previous studies have found that job embeddedness has a negative relationship with the intent to quit a job [79–81]. Quite a few researchers have considered the dimensionalities of job embeddedness and intent to quit (e.g [82]). It was necessary to compare this measurement and the dimensions of job embeddedness. The CFA was conducted on a sample of 433 to confirm the hypothesised five-factor structure using the IBM AMOS.

### Participants

The participants for this study comprised four hundred and thirty-three (433) workers also drawn from two major cities in Nigeria, Enugu and Lagos. The participants comprised of male 240 (57.2%) and 193 (42.8%) female, 203 (49.3%) married and 230 (50.7%) singles.

### Instruments

Expanded multidimensional Turnover Intention Scale (EMTIS). The EMTIS comprised the 25 items developed. The response is patterned on a five-point Likert-type format ranging from 1= (strongly disagree) to 5 (strongly agree)’. The respondents are expected to indicate how much they agree with the listed statements regarding their intention to leave their job. High scores indicate a higher intention of leaving the job, while low scores indicate a lower intention. The TIS has five factors: subjective social status factor (4 items) (e.g., I often feel like quitting this job because my present job position is not compatible with my job resume); Organisational culture factor (3 items) (e.g., My major dissatisfaction in life comes from my job environment); Personal orientation factor (8 items) (e.g., My family is not happy with the nature of my job); Expectation factor (5 items) (e.g., Regardless of the pay, I would prefer working where I will be respected and recognised.); and career Growth factor (5 items) (e.g., I know I deserve a better job, I will go for it when I find one).

Turnover Intention scale (TIS [17]), Turnover intention scale developed by [17] it a six-item scale. Sample items are: ‘How often have you considered leaving your job?‘ and ‘How often do you look forward to another day at work?‘ The authors reported a reliability coefficient of 0.80, while we obtained a reliability coefficient of 0.78 for this study.

Job Embeddedness scale (JES). Job embeddedness was measured by the job embeddedness scale developed by [83]. The 18–item scale assessed how employees are enmeshed in their job. For example, “my job enables me to exploit my skills and talent well”, and “my values and goals are consistent with the company’s values”. The response format of the scale is Likert-type response options, ranging from 1 = strongly disagree to 5 = strongly agree. All the items are directly scored, indicating that the higher the score, the higher the embeddedness; the lower the score, the lower the job embeddedness.

#### Procedure

The three questionnaires were distributed to four states using volunteering colleagues in those states to conduct a wave study. All participants were informed that their responses to the questionnaire would remain confidential and strictly for academic purposes. Most of them voluntarily provided their mobile numbers, which were used to follow up with them on the subsequent questionnaires. Six hundred and eighty (680) copies of the questionnaire were distributed (170) in each of the four states. The questionnaire allocated for each of the four states was (purposively) administered to the employees in the five major sectors as earlier done in the first study. After completion and collection, properly filled copies of the questionnaire were analysed. A total of six hundred and forty-three (643) were returned, twenty-one (21) copies were discarded as a result of improper completion, while six hundred and twenty-two (622) valid copies were used for the analysis, yielding a valid response rate of 91.5% out of six hundred and eighty (680) copies of the questionnaire that were initially distributed.

#### Design/statistics

Using a 3-wave study design, SPSS 25 was used to measure the descriptive and convergent validity of the EMTI scale, turnover intentions scale, and job embeddedness. IBM SPSS AMOS 24 Software was used to test for the CFA and goodness-of-fit statistics, while SmartPLS 3.3 was used for the discriminant validity.

### Result

The CFA of the EMTI scale assessed the significance of the questionnaire constructed from the result of the EFA. The maximum likelihood estimation was used to assess the model fit according to the covariance matrix of the confirmatory data set. The Chi-square (x2) value, the root mean square error of approximation (RMSEA), and the standardised root mean square residual (SRMR) were calculated. Supplementing these indices, we examined model fit by using the comparative fit index (CFI), goodness-of-fit index (GFI), and normed fit index (NFI) [84]. Generally, the criterion for establishing model fit suggests that CFI, GFI, and NFI values close to 0.90 represent an acceptable fit, and 0.90 or higher indicates a good fit [85, 86].

**Fig. 1:**
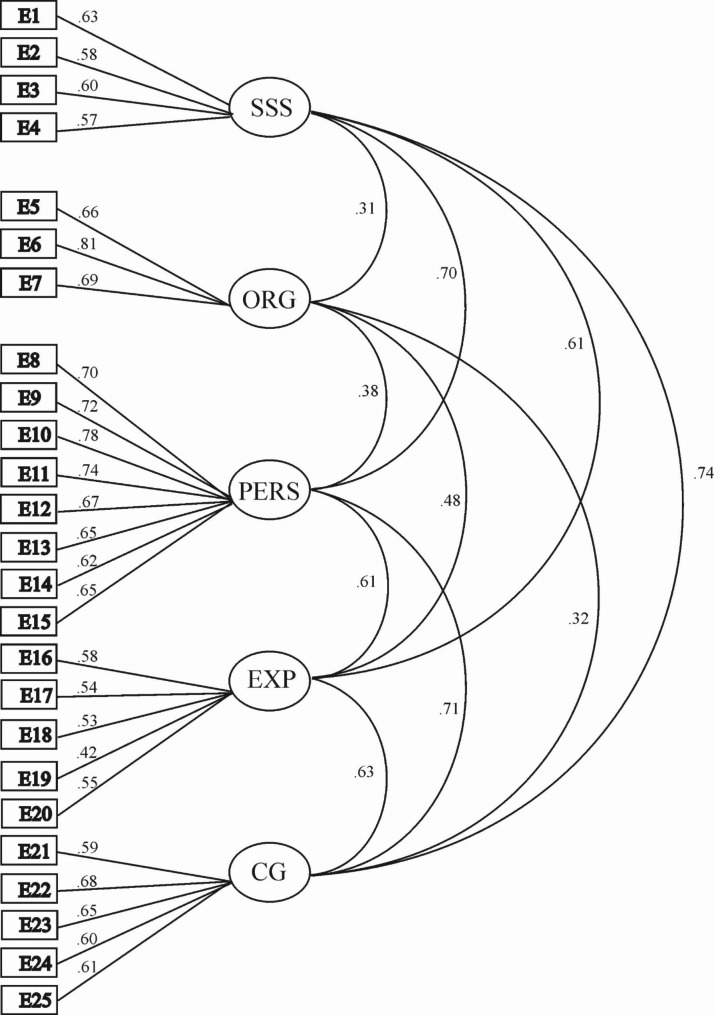
Hierarchical model and factor loadings resulting from confirmatory factor analysis Note: SSS= Subjective Social Status; ORG= Organisational culture; PERS= Personal Orientation; EXP= Expectation; CG= Career Growth

Conducting CFA on the 25 items identified in the EFA and comparing different models (One, uncorrelated, correlated and hierarchical factors, see Table 2; Fig. 1). The correlated and hierarchical five factors produced much better goodness-of-fit statistics than the uncorrelated model.

Convergent validity was established by assessing the patterns of correlation among factors [87, 88]. recommended that convergent validity be evaluated by inspecting the factor loadings, which should be statistically significant and greater than 0.5. The result showed that the dimensions of EMTI related significantly with Bothma and Roodt’s Turnover Intension scale (see Table 3). Also, a divergent validity was done to establish a negative relationship with job embeddedness. Job embeddedness has been used to establish employee retention. Therefore, a negative relationship is expected [83]. The finding was significant as the five dimensions of EMTI had significant negative relationships with the three dimensions of the job embeddedness scale (fit, link, and sacrifice) (see Table 3).

To further solidify the validity of our constructs, a rigorous discriminant validity assessment was undertaken using the Heterotrait-Monotrait (HTMT) Ratio of Correlation. Notably, Henseler et al. [89] and other researchers [90, 91] have emphasised the importance of considering model set-up and desired conservativeness when choosing an HTMT cut-off criterion. Considering this and recognising the theoretical commonalities between the constructs examined in our scale, we opted for a more liberal cut-off value. We set our threshold at 0.90, aligning with Henseler et al.‘s recommendations, especially when constructs share theoretical underpinnings and might be challenging to distinguish in varied research settings. This approach ensures that while we recognise the uniqueness of each construct, we’re also cognizant of their inherent interconnectedness. The results, as detailed in Table 4, reinforce the robustness of our scale, with constructs demonstrating strong intra-construct relations, solidifying the discriminant validity of our measures.

**Table 2:** Confirmatory Factor Analysis of the MTI Scale

Model	${\varvec{x}}^{2}$	df	${\varvec{x}}^{2}$/df	GFI	NFI	CFI	RMSEA	SRMR
Null	2110.43***	364	8.489	0.781	0.796	0.810	0.101	0.3022
One factor	1751.43***	237	7.390	0.792	0.784	0.806	0.102	0.0745
Uncorrelated factors	1724.33***	250	6.897	0.796	0.787	0.811	0.098	0.2771
Correlated factors	576.862***	225	2.564	0.930	0.929	0.955	0.050	0.0407
Hierarchical	680.450***	184	3.698	0.910	0.916	0.936	0.061	0.0504

Note : * p < .05; ** p < .01; *** p < .001

**Table 3:** Convergent and Divergent validity of the EMTI with turnover intention scale and job embeddedness

		1	2	3	4	5	6	7	8	9
1	SSS	--								
2	Organisational culture	0.25^*^	--							
3	Personal orientation	0.54^**^	0.35^**^	--						
4	Expectation	0.50^**^	0.38^**^	0.57^**^	--					
5	Career Growth	0.59^**^	0.25^**^	0.57^**^	0.51^**^	--				
6	TIS	− 0.58**	0.69^**^	0.50^**^	0.54**	0.61**	--			
7	Fit (JE)	− 0.36**	− 0.12^*^	− 0.10^*^	− 0.44**	− 0.36**	− 0.27**	--		
8	Link (JE)	− 0.27**	− 0.39**	− 0.22**	− 0.41^**^	− 0.34*^*^	− 0.21^**^	− 0.66^**^	--	
9	Sacrifice (JE)	− 0.32^*^	− 0.51**	− 0.11^**^	− 0.31**	− 0.50**	− 0.06	− 0.66^**^	− 0.69^**^	--

NOTE: * = p < .05; ** =p < .01; SSS = Subjective Social Status; TIS = Turnover intentions (by Bothma & Roodt, 2013); Fit (JE) = Job Embeddedness (fit dimension); Link (JE) = Job Embeddedness (Link dimension); Sacrifice (JE) = Job Embeddedness (sacrifice dimension)

**Table 4:** HTMT

		1	2	3	4	5
1	Subjective social status	0.88				
2	Organisational culture	0.44	0.85			
3	Personal orientation	0.64	0.38	0.83		
4	Expectation	0.76	0.45	0.61	0.86	
5	Career Growth	0.85	0.38	0.60	0.74	0.88

**Table 5:** Means (SD) MTI scores by the gender

Scales	Total	Male	Female	*t*	*P*
Subjective social Status	12.31(4.25)	12.23(4.18)	12.43(4.35)	− 0.585	0.558
Organisational culture	7.30(3.24)	7.31(3.12)	7.29(3.39)	0.092	0.926
Personal orientation	15.65(5.09)	15.68(4.90)	15.61(5.32)	0.154	0.878
Expectation	12.64(4.42)	12.43(4.36)	12.85(4.50)	-1.189	0.237
Career Growth	12.64(4.20)	12.66(4.15)	12.61(4.28)	0.162	0.729
Total	60.53(16.12)	60.33(15.71)	60.79(16.67)	− 0.346	0.729

### Reported expanded multidimensional turnover intention

As shown in Table 5, the overall mean score of EMTI scale was 60.53 (SD = 16.12). Analysing the five dimensions, it showed that overall EMTI for subjective social status is mean ± SD = 12.23 ± 4.18, organisational culture is mean ± SD = 7.30 ± 3.24, personal orientation is mean ± SD = 15.65 ± 5.09, and expectation is mean ± SD = 12.64 ± 4.20. Finally, the total score on EMTI difference between males and females was not significant (*t* = − 0.3.46, *p* = .73), the same as the five dimensions.

## Discussion

Turnover intention scales have long been the barometer for gauging employee satisfaction in the academic and corporate realms. Yet, while valuable, many of these scales are riddled with methodological pitfalls. These range from concerns over their validity to their often-narrow focus. Within this context, the Expanded Multidimensional Turnover Intentions Scale (EMTIS) emerges as a beacon of innovation. Unlike its predecessors, which tend to provide a singular, limited perspective, EMTIS offers a panoramic view of turnover, encompassing the diverse factors that may influence an individual’s intention to depart.

This multidimensional approach is more than just a structural enhancement; it underscores the intricate landscape of turnover intentions. EMTIS challenges the convention of viewing turnover as a monolithic entity by fragmenting this complex phenomenon into multiple dimensions. Such a revelation reshapes our theoretical understanding, painting a mosaic of factors that come into play. At its core, EMTIS suggests that turnover intentions do not arise from a singular source but are the culmination of various intertwined factors. This perspective aligns with broader shifts in social and organisational psychology, recognising the multifaceted nature of human behaviour. By encompassing dimensions like subjective social status and organizational culture, the scale emphasises that turnover decisions are deeply personal and influenced by many internal and external factors. Armed with this new perspective, researchers find themselves in an expanded field filled with nuanced avenues to explore.

Yet, the implications of EMTIS are not confined to academic corridors. On the practical front, it provides organisations with a granular diagnostic tool. No longer do they need to rely on vague understandings. With EMTIS, they gain unprecedented clarity on the specific issues at play, be it discrepancies in organisational culture, unmet personal expectations, or other underlying causes. Such precision is transformative. It allows organisations to tailor their interventions, addressing the root causes and fostering an environment conducive to employee retention.

Furthermore, EMTIS’s versatility stands as a testament to its potential. Beyond its immediate use as a diagnostic tool, it offers a roadmap for organisations and researchers. Each scale dimension, rich in its insights, beckons further exploration. How might these factors manifest across different sectors? Is there a notable interplay between them? These are just some of the myriad questions that can pave the way for enriched academic discourse.

However, as with any tool developed within a specific cultural context, EMTIS faces the inevitable question of its global adaptability. Can its constructs resonate universally, or do they take on different hues across cultures? The answer to this isn’t merely academic; it has far-reaching implications. For EMTIS to be truly global, it must navigate these cultural nuances, demanding rigorous validation to ensure its dimensions remain relevant across the board. While EMTIS offers a fresh lens on turnover intentions, its journey, especially on the global stage, is just beginning.

### Practical implication

Voluntary turnover has been found to affect organisations negatively, ranging from losing the best heads and hands to the cost of retraining and replacing them. Job retention is a big deal and significantly has implications for the success of organisations. This measure highlights neglected areas like social status, organisational culture, psychological expectations, room for career growth, and personal orientation preferences. This measure further goes to help the management of human resource personnel of organisations to look beyond mere incentives as the major reason employees quit. Frequent evaluation of employees to sense areas the organisation can improve on could help the organisation keep their best hands than being taken unaware of their actual turnovers.

### Limitations

Notwithstanding the contributions of this study, some limitations were noticed. With all the effort to build confidence and trust with the respondents that the aim of the study was for educational purposes, we cannot say there was no fear on the part of the respondents that the organisation might see their responses. Secondly, some factors (e.g., organisational culture) with fewer items have not exhaustively covered the organisational culture. For future research, an improvement in a revalidation study can make it better. Finally, the data collection technique was cross-sectional, and it has limitations, such as the difficulty of making causal statements and its susceptibility to biases like non-response and recall bias [92].

## Conclusions

This study’s major goal was to broaden the understanding and application of the turnover intentions construct by considering the construct’s multi-dimensionality. This study has established that the new measure is a reliable and valid instrument to assess the multiple dimensions of turnover intention.

## Data Availability

The datasets generated and/or analysed during the current study are not publicly available due to privacy restrictions but are available from the corresponding author on reasonable request.

## References

[1] Hausknecht JP, Holwerda JA. When Does Employee Turnover Matter? Dynamic Member Configurations. Productive Capacity, and Collective Performance. 24, 210–225 (2013).

[2] Hom PW, Lee TW, Shaw JD, Hausknecht JP (2017). One hundred years of employee turnover theory and research. J Appl Psychol.

[3] Ajzen I (1991). The theory of planned behavior. Organ Behav Hum Decis Process.

[4] Vardaman JM (2015). Translating intentions to behavior: the interaction of network structure and behavioral intentions in understanding employee turnover. Organ Sci.

[5] Menezes I, Lozado J, Menezes AC, Moraes E, Sandbrand D, Muszynski P, Ruggeri K. Development and validation of the multidimensional turnover intentions scale. In BAM2018 Conference Proceedings: Driving productivity in uncertain and challenging times (2018).

[6] Hanushek EA, Rivkin SG (2007). Pay, working conditions, and teacher quality. Future Child.

[7] Sharma S, Sharma SK (2016). Team Resilience: Scale Development and Validation. Vision.

[8] March JG. In: Simon HA, editor. Organisations. Wiley; 1958.

[9] Price J (1977). The study of turnover.

[10] Lee TW, Mitchell TR (1994). An alternative approach: the unfolding model of voluntary employee turnover. Acad Manag Rev.

[11] Mitchell TR (2001). Why people stay: using job embeddedness to predict voluntary turnover. Acad Manag J.

[12] Griffeth RW, Hom PW, Gaertner SA (2000). Meta-analysis of antecedents and correlates of employee turnover: update, moderator tests, and research implications for the next millennium. J Manag.

[13] Holtom BC, Mitchell TR, Lee TW, Eberly MB (2008). Turnover and Retention Research: a glance at the past, a closer review of the Present, and a venture into the future. Acad Manag Ann.

[14] Bluedorn AC. The theories of turnover: causes, effects, and meaning. In: Bacharach S, editor. Perspectives in Organisational sociology: theory and research. JAI Press; 1982.

[15] Hom PW, Griffeth RW (1991). Structural equations modeling test of a turnover theory: cross-sectional and longitudinal analyses. J Appl Psychol.

[16] Vigoda E, Organisational Politics (2000). Job attitudes, and work outcomes: exploration and implications for the Public Sector. J Vocat Behav.

[17] Bothma CFC, Roodt G (2013). The validation of the turnover intention scale. SA J Hum Resour Manag.

[18] Ghosh P, Satyawadi R, Prasad Joshi J, Shadman M (2013). Who stays with you? Factors predicting employees’ intention to stay. Int J Organ Anal.

[19] Jackman MR, Jackman RW (1973). An interpretation of the relation between objective and subjective social status. Am Sociol Rev.

[20] Singh-Manoux A, Adler NE, Marmot MG (2003). Subjective social status: its determinants and its association with measures of ill-health in the Whitehall II study. Soc Sci Med.

[21] Diemer MA (2013). Best practices in conceptualizing and measuring social class in psychological research. Arch Soc Issues Public Health Policy.

[22] Feng D, Su S, Yang Y, Xia J, Su Y (2017). Job satisfaction mediates subjective social status and turnover intention among chinese nurses. Nurs Health Sci.

[23] Joo B-K (Brian), Hahn H-J, Peterson SL Turnover intention, editors. the effects of core self-evaluations, proactive personality, perceived organizational support, developmental feedback, and job complexity. *Hum. Res. Dev. Int.18*(2), 116–130 (2015).

[24] Ali N, Baloch QB (2010). Impact of job satisfaction on turnover intention: an empirical evidence. J Manag Sci.

[25] Chen G (2021). Current status and related factors of turnover intention of primary medical staff in Anhui Province, China: a crosssectional study. Hum Resour Health.

[26] He R, Liu J, Zhang W-H (2020). Turnover intention among primary health workers in China: a systematic review and meta-analysis. BMJ Open.

[27] Lu Y et al. Analyzing the stability of rural doctor team in Shandong Province in different dimensions. Health Care Manag Sci 517–20 (2018).

[28] MacLeod J, Davey SG, Metcalfe C, Hart C (2005). Is subjective social status a more important determinant of health than objective social status? Evidence from a prospective observational study of scottish men. Soc Sci Med.

[29] Tharp MB. Four Organisational Culture Types. http://faculty.mu.edu.sa/public/uploads/1360757023.3588organisational cult98.pdf (2009).

[30] Hakim A (2015). Effect of Organisational Culture, Organisational Commitment to performance: study in hospital of District South Konawe of Southeast Sulawesi. Int J Eng Sci.

[31] Dwivedi S, Kaushik S, Luxmi (2014). Impact of Organisational Culture on turnover intentions in BPO Sector in India. Indian J Ind Relat.

[32] Enwereuzor IK, Ugwu LE (2021). Clarifying the interface between respectful leadership and intention to stay. J Health Organ Manag.

[33] Jacobs EJ, Roodt G. The mediating effect of knowledge sharing between organisational culture and turnover intentions of professional nurses. SA J. Inf.

[34] Kee KN. The Relationship between Selected Organisational Culture and Employees’ Turnover Intentions. (Bachelor Thesis). Retrieved from ir. unimas.my/6786/1/ (2010).

[35] Firth L, Mellor DJ, Moore KA, Loquet C (2004). How can managers reduce employee intention to quit?. J Manag Psychol.

[36] Parker DF, DeCotiis TA (1983). Organisational determinants of job stress. Organ Behav Hum Perform.

[37] Sawyerr OO, Srinivas S, Wang S (2009). Call center employee personality factors and service performance. J Serv Mark.

[38] Shih-Tse WE (2014). The effects of relationship bonds on emotional exhaustion and turnover intentions in frontline employees. J Serv Mark.

[39] Kim MJ, Han SS (2007). Comparison of job satisfaction, commitment to organisation, nursing organisation culture and job experience between national/public hospital nurses and private hospital nurses. J East-West Nurs Res.

[40] Bakkal E, Serener S, Myrvang NA (2019). Toxic leadership and turnover intention: mediating role of job satisfaction. Rev Cercetare Intervent Soc.

[41] Goldman A (2011). Demagogue to dialogue: an alternative to toxic leadership incorporate downsizings. Organ Dyn.

[42] Ugwu FO, Onyishi EI, Anozie OO, Ugwu LE (2021). Customer incivility and employee work engagement in the hospitality industry: roles of supervisor positive gossip and workplace friendship prevalence. J Hosp Tour Insights.

[43] Abdollahzadeh F (2017). How to prevent workplace incivility? Nurses’ perspective. Iran J Nurs Midwifery Res.

[44] Doty J, Fenlason J. Narcissism and toxic leaders. Mil Rev (January-February), 55–60 (2013). http://www.dtic.mil/get-tr-doc/pdf?AD=ADA576059

[45] Taştan SB, Environment (2017). In search for the toxic Behaviours in Organisations with a research in Healthcare. Postmod Open.

[46] Ali Shah I, Fakhr Z, Ahmad MS, Zaman K (2010). Measuring push, pull and personal factors affecting turnover intention: a case of university teachers in Pakistan. Rev Econ Bus Stud.

[47] Muchinsky PM, Tuttle ML (1979). Employee turnover: an empirical and methodological assessment. J Vocat Behav.

[48] Babajide EO (2010). The influence of personal factors on workers’ turnover intention in work organisations in South-West Nigeria. J Divers Manag.

[49] Elliott B, Crosswell L. Commitment to teaching: Australian perspectives on the interplays of the professional and the personal in teachers’ lives. Paper presented at the International Symposium on Teacher Commitment at the European Conference on Educational Research, Lille, France (2001).

[50] Fried RL. The passionate teacher: a practical guide. Beacon Press; 1995.

[51] Nias J (1996). Thinking about feeling: the emotions in teaching. Camb J Educ.

[52] Chou CK, Chen ML (2016). A qualitative study on perceived value and loyalty: a moderated-mediation framework. Corp Manag Rev.

[53] Liou FM, Tsai YH (2016). Latent trajectories of competitive heterogeneity: bridging the gap in theories between persistent performance and value creation. Corp Manag Rev.

[54] Lin C-H, Sanders K (2017). HRM and innovation: a multi-level organisational learning perspective. Hum Resour Manag J.

[55] Rousseau DM (1995). Psychological contracts in Organisations: understanding Written and Unwritten agreements.

[56] Bravo GA, Won D, Chiu W (2019). Psychological contract, job satisfaction, commitment, and turnover intention: exploring the moderating role of psychological contract breach in National Collegiate athletic association coaches. Int J Sports Sci Coach.

[57] Kanu GC, et al. Psychological contract breach and turnover intentions among lecturers: the moderating role of Organisational Climate. Front Educ. 2022;7. 10.3389/feduc.2022.784166

[58] Moquin R, Riemenschneider CK, Wakefield RL (2019). Psychological contract and turnover intention in the Information Technology Profession. Inf Syst Manag.

[59] Arasli H, Arici HE (2019). Çakmakoğlu Arici, N. Workplace favouritism, psychological contract violation and turnover intention: moderating roles of authentic leadership and job insecurity climate. Ger J Hum Resour Manag.

[60] Neckebrouck J, Schulze W, Zellweger T (2018). Are family firms good employers? Acad. Manag J.

[61] Boudrias V (2020). Investigating the role of psychological need satisfaction as a moderator in the relationship between job demands and turnover intention among nurses. Empl Relat.

[62] Yang C, Chen Y, Roy XZ, Mattila AS (2020). Unfolding deconstructive effects of negative shocks on psychological contract violation, organisational cynicism, and turnover intention. Int J Hosp Manag.

[63] Blau PM. Exchange and Power in Social Life. Wiley; 1964.

[64] Aydogdu S, Asikgil B (2011). An empirical study of the relationship among job satisfaction, Organisational commitment and turnover intention. Int Rev Manag Mark.

[65] Mobley W (1977). Intermediate linkages in the relationship between job satisfaction and employee turnover. J Appl Psychol.

[66] Weng Q, McElroy JC (2012). Organisational career growth, affective occupational commitment and turnover intentions. J Vocat Behav.

[67] Weng Q, McElroy JC, Morrow PC, Liu R (2010). The relationship between career growth and organisational commitment. J Vocat Behav.

[68] Chen JQ (2016). The role of career growth in chinese new employee’s turnover process. J Career Dev.

[69] Albrecht S (2006). Predictors of employee extra-role performance and turnover intentions in the public sector: an integrated model. Int J Hum Resour Dev Manag.

[70] Griek OHV, Clauson MG, Eby LT (2018). Organisational career growth and proactivity. A typology for individual career development. J Career Dev.

[71] Tsui AS, Pearce JL, Porter LW, Tripoli AM (1997). Alternative approaches to the employee-organisation relationship: does investment in employees pay off? Acad. Manag J.

[72] Yusoff MSB (2019). ABC of content validation and content validity index calculation. Educ Med J.

[73] Trochim WMK. The Qualitative Debate. Research Methods Knowledge Base. http://www.socialresearchmethods.net/kb/qualmeth.php (2007).

[74] Worthington RL, Whittaker TA (2006). Scale Development Research: a content analysis and recommendations for best Practices. Couns Psychol.

[75] Hair JF, Black WC, Babin BJ, Anderson R. E. Multivariate data analysis: Pearson new international edition. Pearson; 2014.

[76] Raine-Eudy R (2000). Using structural equation modeling to test for differential reliability and validity: an empirical demonstration. Struct Equ Model.

[77] Fornell CG, Larcker DF (1981). Evaluating structural equation models with unobservable variables and measurement error. J Mark Res.

[78] Krabbe PFM, Validity. The measurement of health and health status. Academic Press; 2017. pp. 113–34.

[79] Clinton M, Knight T, Guest DE (2012). Job embeddedness: a new attitudinal measure. Int J Sel Assess.

[80] Dechawatanapaisal D (2018). The moderating effects of demographic characteristics and certain psychological factors on the job embeddedness-turnover relationship among thai healthcare employees. Int J Organ Anal.

[81] Karatepe OM (2013). The effects of work overload and work-family conflict on job embeddedness and job performance. Int J Contemp Hosp Manag.

[82] Fuchs RM, Links (2021). Fit or sacrifice: job embeddedness and intention to quit among Generation Y. Eur J Manag Bus Econ.

[83] Nafei W (2014). The role of Organisational DNA in improving Organisational performance: a study on the Industrial Companies in Egypt. Int Bus Res.

[84] Browne MW, Cudeck R. Alternative ways of assessing model fit. In: Bollen KA, Long JS, editors. Testing structural equation models. Sage; 1993. pp. 136–62.

[85] Faulkner J, Laschinger H (2007). The effects of structural and psychological empowerment on perceived respect in acute care nurses. J Nurs Manag.

[86] McCoach DB, Gable RK, Madura JP. Instrument development in the affective domain: School and corporate applications. 3rd ed. Springer Science + Business Media; 2013.

[87] Tabachnick BG, Fidell LS (2014). Using multivariate statistics.

[88] Malhotra NK, Introduction. Analyzing Accumulated Knowledge and Influencing future Research. In: Malhotra, N.K, editor Review of Marketing Research (Review of Marketing Research, Vol. 7). Emerald Group Publishing Limited, Bingley, pp. xiii-xxviii (2010).

[89] Henseler J, Ringle CM, Sarstedt M (2015). A new criterion for assessing discriminant validity in variance-based structural equation modelling. J Acad Mark Sci.

[90] Franke G, Sarstedt M (2019). Heuristics versus statistics in discriminant validity testing: a comparison of four procedures. Internet Res.

[91] Roemer E, Schuberth F, Henseler J (2021). HTMT2–an improved criterion for assessing discriminant validity in structural equation modelling. Ind Manag Data Syst.

[92] Wang D (2019). A test of psychology of working theory among chinese urban workers: examining predictors and outcomes of decent work. J Vocat Behav.

